# The role of good governance in the race for global vaccination during the COVID-19 pandemic

**DOI:** 10.1038/s41598-021-01831-0

**Published:** 2021-11-17

**Authors:** Moosa Tatar, Mohammad Reza Faraji, Jalal Montazeri Shoorekchali, José A. Pagán, Fernando A. Wilson

**Affiliations:** 1 Matheson Center for Health Care Studies, University of Utah, 295 Chipeta Way, Salt Lake City, UT 84108 USA; 2 Department of Population Health Sciences, University of Utah, Salt Lake City, UT USA; 3 Department of Computer Science and Information Technology, Institute for Advanced Studies in Basic Sciences (IASBS), 444 Sobouti Blvd, Zanjan, Iran; 4 Institute for Humanities and Cultural Studies, Tehran, Iran; 5 Department of Public Health Policy and Management, School of Global Public Health, New York University, 715/719 Broadway 10th Fl., New York, NY 10003 USA

**Keywords:** Natural hazards, Disease prevention, Health policy, Public health, Infectious diseases, Vaccines

## Abstract

Governments have developed and implemented various policies and interventions to fight the COVID-19 pandemic. COVID-19 vaccines are now being produced and distributed globally. This study investigated the role of good governance and government effectiveness indicators in the acquisition and administration of COVID-19 vaccines at the population level. Data on six World Bank good governance indicators for 172 countries for 2019 and machine-learning methods (K-Means Method and Principal Component Analysis) were used to cluster countries based on these indicators and COVID-19 vaccination rates. XGBoost was used to classify countries based on their vaccination status and identify the relative contribution of each governance indicator to the vaccination rollout in each country. Countries with the highest COVID-19 vaccination rates (e.g., Israel, United Arab Emirates, United States) also have higher effective governance indicators. Regulatory Quality is the most important indicator in predicting COVID-19 vaccination status in a country, followed by Voice and Accountability, and Government Effectiveness. Our findings suggest that coordinated global efforts led by the World Health Organization and wealthier nations may be necessary to assist in the supply and distribution of vaccines to those countries that have less effective governance.

## Introduction

By the end of March 2021, more than 129 million COVID-19 cases and 2.8 million COVID-19 deaths have been reported globally, and new COVID-19 variants threaten to strengthen this upward trend in cases and deaths^1^. From the beginning of the pandemic, the World Health Organization (WHO) and other institutions partnered in responding to COVID-19 by tracking the pandemic, advising on critical interventions, and distributing vital medical supplies^2^. However, the best way to control and stop the COVID-19 pandemic as quickly as possible is to develop and deploy safe and effective COVID-19 vaccines and immunize a sufficiently large share of the world to attain herd immunity^3^.

**Figure 1:**
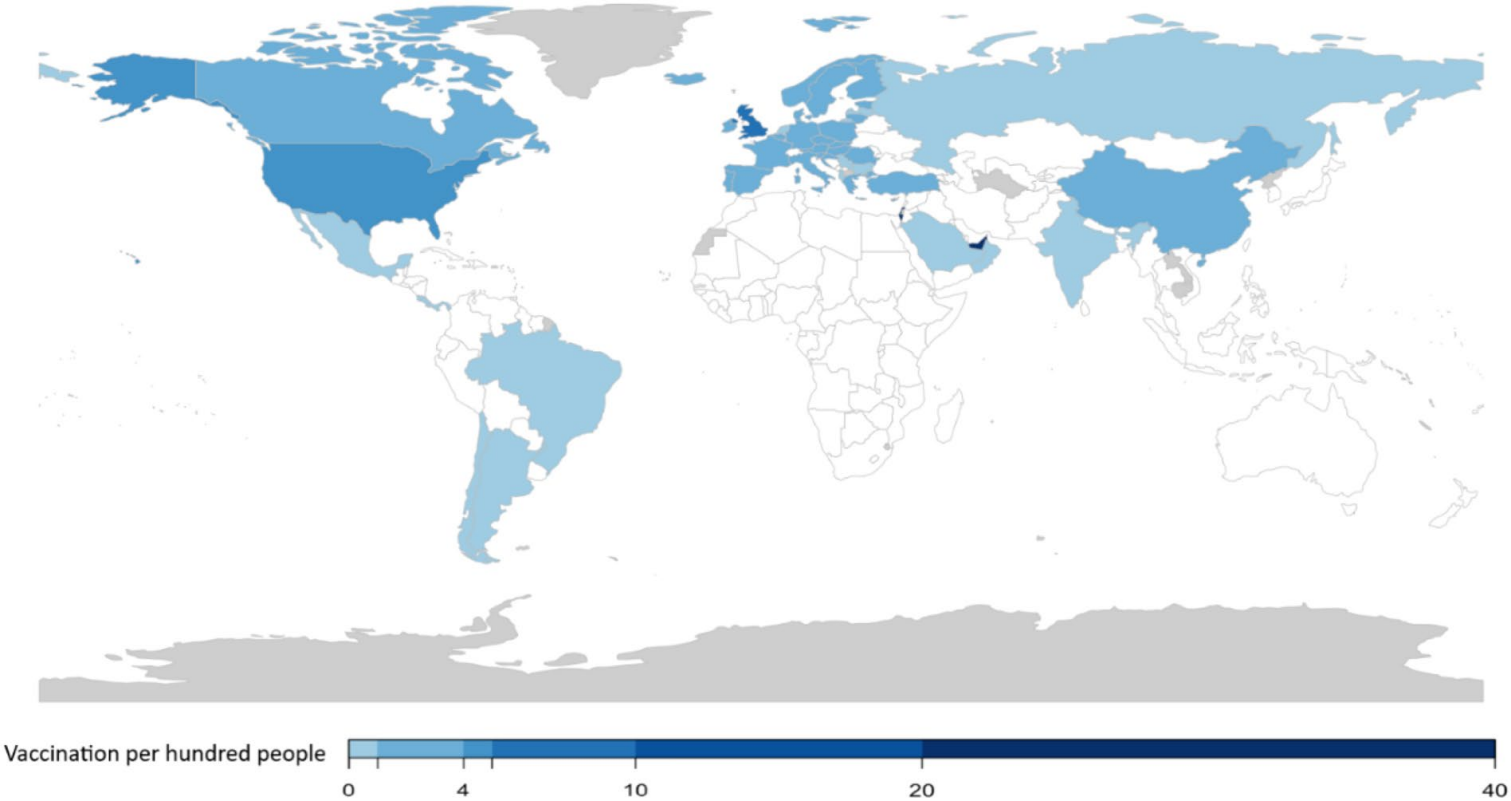
COVID-19 Vaccination per Hundred People Approximately One Month After the First Public COVID-19 Vaccination (by January 20, 2021). Darker blue shows higher vaccination rates per hundred people. Countries with no available data are shown in gray. The maps were generated using RStudio 4.0.2 (R Core Team, 2020). Downloaded from: https://www.rstudio.com/products/rstudio/download/.

**Figure 2:**
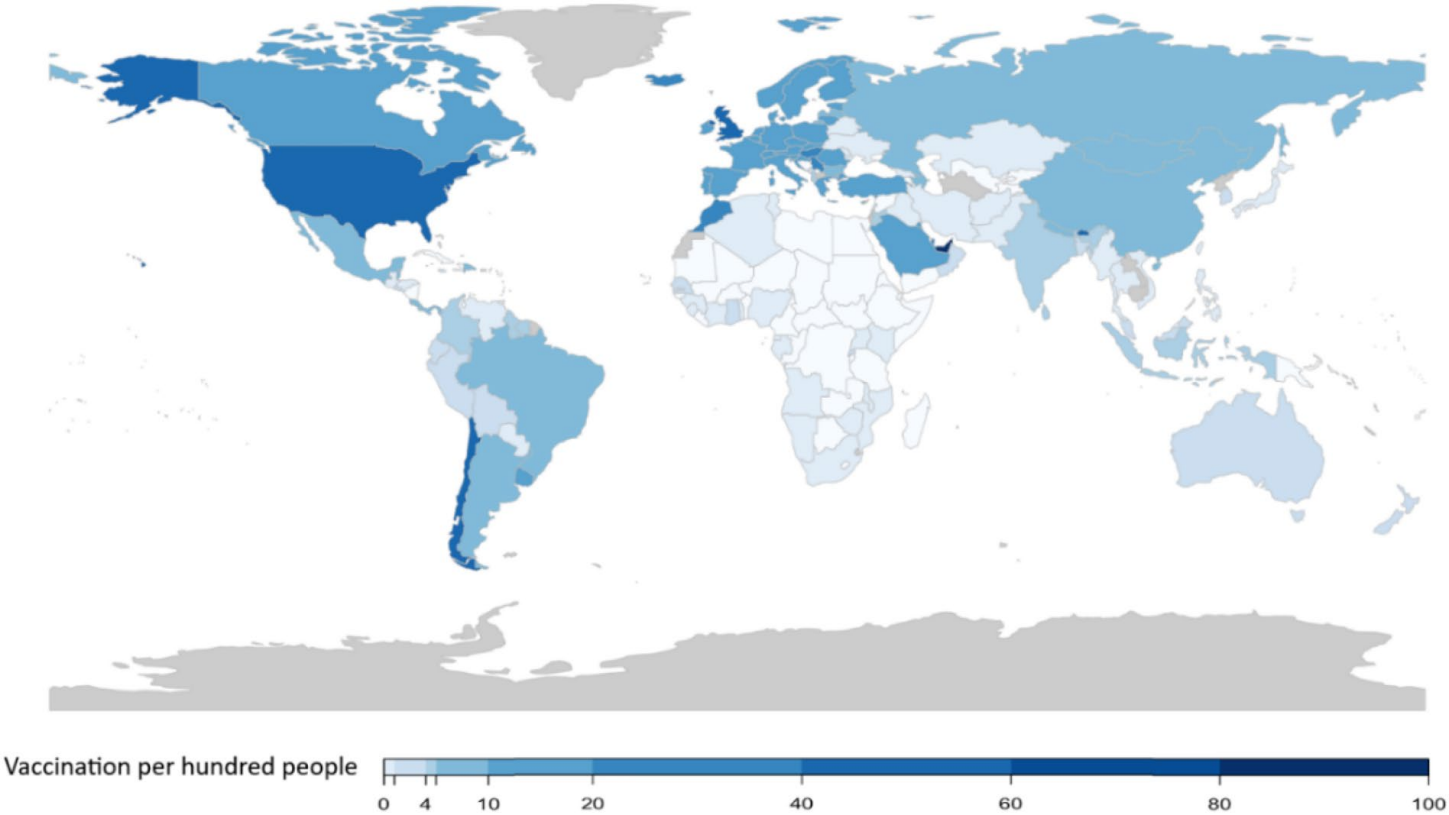
COVID-19 Vaccination per Hundred People Approximately Three Months After the First Public COVID-19 Vaccination (by March 31, 2021). Darker blue shows higher vaccination rates per hundred people. Countries with no available data are shown in gray. The maps were generated using RStudio 4.0.2 (R Core Team, 2020). Downloaded from: https://www.rstudio.com/products/rstudio/download/.

As the COVID-19 pandemic started, scientists emphasized the need to rapidly develop a vaccine against COVID-19 largely by utilizing emerging technologies in genomics and structural biology^4^. Approximately one year after the pandemic started, various COVID-19 vaccines (e.g., Pfizer/BioNTech, Moderna, AstraZeneca, Johnson & Johnson, Sinopharm) are being produced and globally distributed. The first COVID-19 vaccinations outside of clinical trials began in early December 2020 in the United Kingdom and, by January 20, 2021, more than 54 million people were vaccinated globally^5^. The health and economic benefits of wide COVID-19 vaccination efforts are very high, especially if vaccines are deployed quickly to prevent repeated waves of COVID-19 cases and the emergence of new variants^6^. However, the distribution of COVID-19 vaccines has varied widely. Some countries such as Israel and the United Arab Emirates have vaccinated a substantial share of their population. On the other hand, several countries have not even started a COVID-19 vaccination campaign (see Figs. 1 and 2).

The role of governments in controlling the COVID-19 pandemic is crucial^7,8^. Worldwide, national governments have allocated extensive financial and medical resources to fight the COVID-19 pandemic. Additionally, many countries have implemented various policies and restrictions and deployed a wide range of nonpharmaceutical interventions (i.e., curfews and business closures) to combat COVID-19^7^. Studies have investigated the effectiveness of governments in controlling the COVID-19 pandemic by implementing these nonpharmaceutical interventions and by investigating the role of different policies, COVID-19 testing, and hospital capacity in controlling the pandemic and reducing COVID-19 mortality^8–10^. However, a comprehensive analysis of the role of good governance and indicators of government effectiveness in the purchase and administration of COVID-19 vaccines at the population level has not been conducted, to our knowledge. This study uses data from the World Bank's Worldwide Governance Indicators (WGI), COVID-19 vaccination per-capita rates, and machine learning methods to investigate whether good governance indicators are predictive of COVID-19 vaccination rates^11^.

## Methods

### Study setting, data and design

Governance indicators for 2019 (the most recent data available) and COVID-19 vaccination data were used to build a dataset with 172 countries. Our ecological study included data on six governance indicators and COVID-19 vaccination rates, COVID-19 cases, and COVID-19 deaths. The WGI is available from the World Bank^11^, and includes governance-related indicators in six areas: voice and accountability, political stability and absence of violence, government effectiveness, regulatory quality, rule of law, and control of corruption (see Appendix Table A1 for definitions). From the date that the first person was vaccinated against COVID-19 (December 8, 2020, after the United Kingdom approved the first COVID-19 vaccine for its population), we used data up to January 20, 2021, which gave a window of nearly six weeks to investigate the performance of countries at the beginning of the global COVID-19 vaccine race. Data on COVID-19 were obtained from the Our World in Data website, which collates and reports data obtained from official government sources for each country providing data^12^.

We explored good governance's role in purchasing and administering the COVID-19 vaccine using machine learning methods, and we conducted cluster analysis for the 172 countries. Afterward, we analyzed each cluster based on their good governance indicators and COVID-19 vaccination rates and status. Finally, using the XGBoost methodology, we classified countries based on their vaccination status (no vaccinations = 0, vaccination started = 1) and used six good governance indicators to predict their vaccination status. Institutional review board approval was not needed as no patients were involved in the study.

### Statistical analysis

For the first analysis, we clustered the good governance data using the K-means clustering method. This method partitions data into K clusters by defining each cluster based on minimizing the sum of the squared distance of good governance data points with their mean value^13^. We used Principal Components Analysis (PCA) and projected the good governance data onto the first two principal components to visualize the clusters in two dimensions (good governance data have six dimensions, and we needed to decrease the dimensions down to two dimensions for visualization)^14^. After clustering countries based on their good governance, we analyzed the results.

For the second analysis, we used the XGBoost machine learning method and classified countries based on their vaccination status (i.e., no vaccination = 0, vaccination started = 1). XGBoost is an efficient algorithm that delivers high performance and accuracy^15^. We employed several evaluation metrics such as accuracy, sensitivity, specificity, and F1-score to assess the classifier accuracy at predicting the class label of countries that have started vaccination (i.e., those labeled "1")^16^. Sensitivity denotes the proportion of countries that have started the COVID-19 vaccination and predicted correctly as class 1 (vaccination status is labeled "1"). Specificity is the proportion of countries that have not started the COVID-19 vaccination and are predicted correctly as class 0 (vaccination status is labeled "0"). Precision is the number of countries correctly identified as countries that started vaccination out of entire countries that have been predicted to start their vaccination. F1-Score is a harmonic mean of precision and recall (which is equal to sensitivity measure). We also reported the Confusion Matrix, which describes the binary classification prediction results.

Additionally, using XGBoost Gain relative importance, we identified the relative contribution of each governance factor to our model that predicts the vaccination status for each country. Gain is the most relevant attribute to interpret the results. Higher Gain implies higher importance in generating the prediction (i.e., the good governance factor's contribution in explaining vaccination status). Finally, we conducted additional sensitivity analyses using data up to March 31, 2021, which gave a window of approximately three months to investigate the performance of countries amid the global COVID-19 vaccine race. The analyses were conducted in the year 2021 using RStudio 4.0.2 (R Core Team, 2020).

### Patient and public involvement statement

No patients were involved in this research, and the article does not involve human participants and does not contain personal medical information.

## Results

Table A2 provides sample characteristics (descriptive statistics) of the good governance indicators and COVID-19 in the 172 countries included in our study. On average, 19,486 COVID-19 cases per million were reported by January 20, 2021, in the study sample, and a maximum of 120,469 of COVID-19 cases per million people was recorded by that time. Also, on average, 355 COVID-19 deaths per million people and a maximum of 1,775 COVID-19 deaths per million people in a country were reported. Additionally, on average, 0.9 COVID-19 vaccinations per hundred people and a maximum of 36.8 COVID-19 vaccinations per hundred people were reported by January 20, 2021 (see Appendix Table A2 for detailed descriptive statistics).

**Figure 3:**
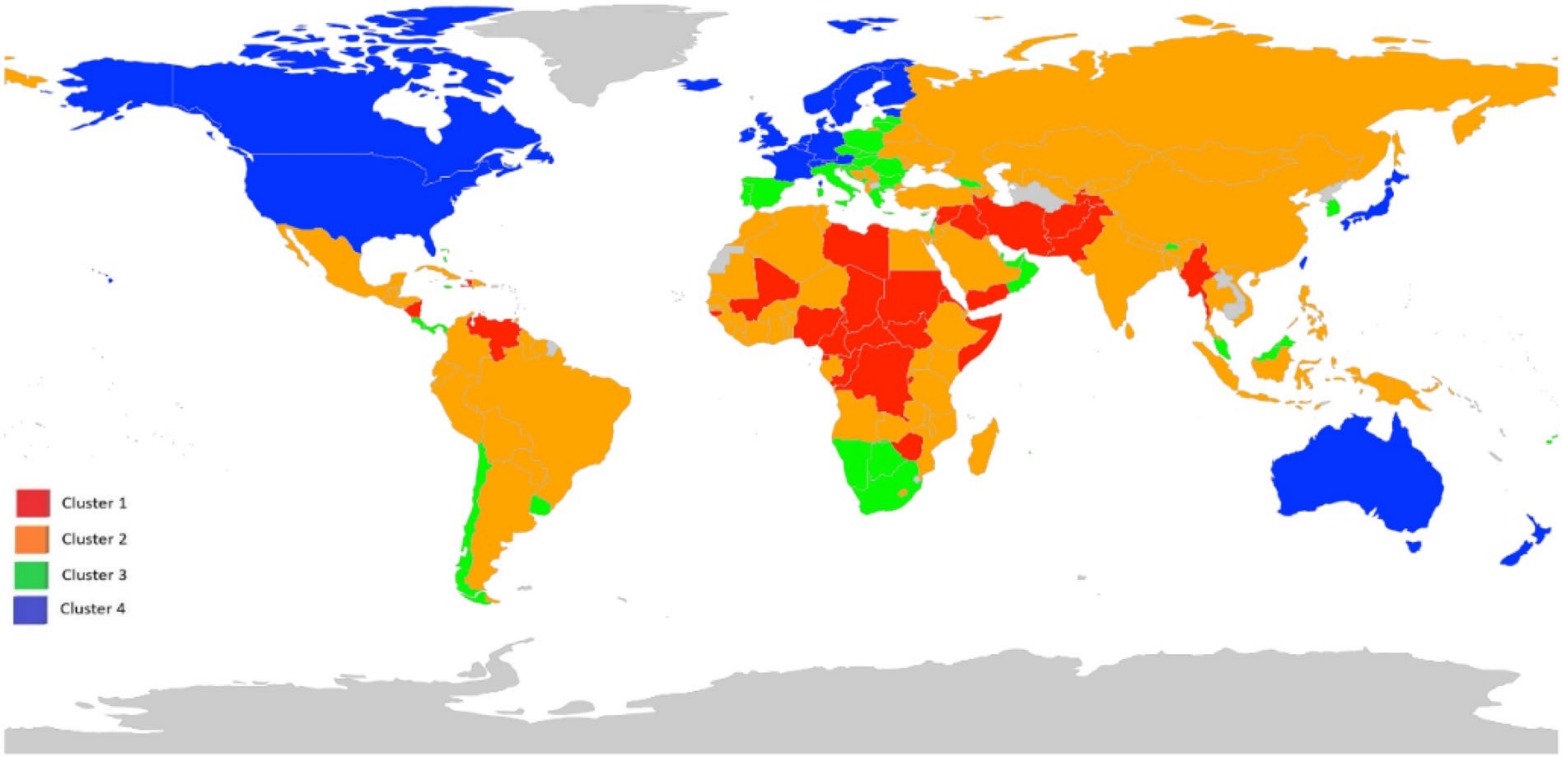
Clustering results of countries' good governance indicators using the K-means clustering algorithm and visualized using principal components analysis. Cluster 1: Afghanistan, Antigua and Barbuda, Burundi, Cameroon, Central African Republic, Chad, Comoros, Congo, Democratic Republic of Congo, Equatorial Guinea, Eritrea, Guinea-Bissau, Haiti, Iran, Iraq, Lebanon, Libya, Mali, Myanmar, Nicaragua, Nigeria, Pakistan, Somalia, South Sudan, Sudan, Syria, Tajikistan, Venezuela, Yemen, Zimbabwe, Cluster 2: Albania, Algeria, Angola, Argentina, Armenia, Azerbaijan, Bahrain, Bangladesh, Belarus, Belize, Benin, Bolivia, Bosnia and Herzegovina, Brazil, Burkina Faso, China, Colombia, Cuba, Djibouti, Dominican Republic, Ecuador, Egypt, El Salvador, Ethiopia, Gabon, Gambia, Ghana, Guatemala, Guinea, Guyana, Honduras, India, Indonesia, Ivory Coast, Jordan, Kazakhstan, Kenya, Kosovo, Kuwait, Kyrgyzstan, Lesotho, Liberia, Madagascar, Malawi, Maldives, Mauritania, Mexico, Moldova, Mongolia, Morocco, Mozambique, Nepal, Niger, North, Macedonia, Papua New Guinea, Paraguay, Peru, Philippines, Russia, Rwanda, Saudi Arabia, Senegal, Serbia, Sierra Leone, Sri Lanka, Suriname, Tanzania, Thailand, Togo, Trinidad and Tobago, Tunisia, Turkey, Uganda, Ukraine, Uzbekistan, Vietnam, ,Zambia, Cluster 3: Bahamas, Barbados, Bhutan, Botswana, Brunei, Bulgaria, Cape Verde, Chile, Costa Rica, Croatia, Cyprus, Czech Republic, Fiji, Georgia, Greece, Grenada, Hungary, Israel, Italy, Jamaica, Latvia, Lithuania, Malaysia, Malta, Mauritius, Montenegro, Namibia, Oman, Panama, Poland, Portugal, Qatar, Romania, Seychelles, Slovakia, Slovenia, South Africa, South Korea, Spain, United Arab Emirates, Uruguay, Cluster 4: Andorra, Australia, Austria, Belgium, Canada, Denmark, Estonia, Finland, France, Germany, Iceland, Ireland, Japan, Liechtenstein, Luxembourg, Netherlands, New Zealand, Norway, Singapore, Sweden, Switzerland, Taiwan, United Kingdom, United States. Countries with no available data are shown in gray. The maps were generated using RStudio 4.0.2 (R Core Team, 2020). Downloaded from: https://www.rstudio.com/products/rstudio/download/.

K-means clustering resulted in four clusters of countries based on their good governance data. Figure 3 depicts the map of clustering results of good governance indicators for each country using the K-means clustering algorithm and visualized using PCA. Good governance data was projected into the first two principal components, containing the most variation in the data (see Appendix Fig. A1). A value of 87.1 in the x-axis (Principal Component 1) implies that the first principal component accounts for 87.1% of good governance variation. The second principal component accounts for 5.2% of the variation. In total, 51 countries out of 172 countries started COVID-19 vaccination approximately one month after the first public COVID-19 vaccination in the United Kingdom. Cluster 1 consists of 30 countries, mainly African and Middle Eastern countries, with poor governance indicators. None of the Cluster 1 countries started COVID-19 vaccinations. Cluster 2 was the largest cluster and consists of 77 countries with a mixture of relatively good and poor governance indicators. Only 11 of the Cluster 2 countries started vaccinations. However, Bahrain, with 8.47% of its population vaccinated, had one of the highest global vaccination rates. Cluster 3 consists of 41 countries with relatively acceptable good governance indicators, and 23 of those countries started COVID-19 vaccination. Israel, the United Arab Emirates, and Seychelles, which had the highest vaccination rates globally, belonged to this group, with vaccination rates of 36.76%, 21.85%, and 13.39%, respectively. Cluster 4 consists of 24 countries and had the highest good governance indicators. Seventeen of the Cluster 4 countries had started the COVID-19 vaccination in our study period. Also, countries with high rates of COVID-19 vaccination, such as the United Kingdom (8.01%) and the United States (4.94%), were in Cluster 4 (see Appendix Table A3).

Table A4 shows the Confusion Matrix and describes the binary classification prediction results. The classifier evaluation metrics, including accuracy (0.81), sensitivity (0.67), specificity (0.87), precision (0.68), F1 (0.67), and balanced accuracy, confirmed the accuracy of the model and the reliability of the predictions (see Appendix Table A5).

**Figure 4:**
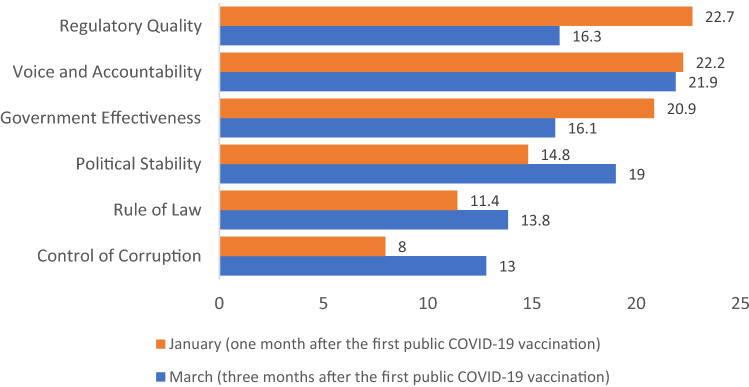
Good Governance Feature (Indicator) Gain Relative Importance. Gain denotes indicator's (feature) relative contribution in explaining variation in outcomes. A higher indicator gain implies greater importance of the indicator for generating a prediction.

Figure 4 presents each good governance indicator's relative importance in predicting the COVID-19 vaccination status based on the XGBoost binary classification model. The regulatory quality in a country is the most important indicator in predicting COVID-19 vaccination status in a country, followed by voice and accountability, and government effectiveness. The relative contributions of these indicators are 22.7%, 22.2%, and 20.9%, respectively. Also, political stability, rule of law, and control of corruption have relative contributions of 14.8%, 11.4%, and 8%, respectively (see Appendix Table A6).

Results of sensitivity analyses using data up to March 2021 shows that, in total, 131 countries out of 172 countries started COVID-19 vaccinations approximately three months (100 days) after the first public COVID-19 vaccination in the United Kingdom. 11 of the Cluster 1 countries (out of 30 countries) started COVID-19 vaccinations. In Cluster 2, which is the largest cluster (77 countries), 58 countries started vaccinations. 38 countries of Cluster 3 started COVID-19 vaccination. Israel, Seychelles, and the United Arab Emirates (within Cluster 3) still had the highest vaccination rates globally, with vaccination rates of 116%, 102%, and 84%, respectively. All of the 24 countries of Cluster 4 that had the highest good governance indicators had started COVID-19 vaccinations approximately three months after the first public COVID-19 vaccination.

Figure 4 also presents each good governance indicator's relative importance in predicting COVID-19 vaccination status based on the XGBoost binary classification model approximately three months after first COVID-19 vaccine. The voice and accountability in a country is the most important indicator in predicting COVID-19 vaccination status in a country, followed by political stability, and regulatory quality, with relative gains of 21.9%, 19%, and 16.3%, respectively. Additionally, government effectiveness, rule of law, and control of corruption have relative contributions of 16.1%, 13.8%, and 13%, respectively (see Appendix Table A6).

## Discussion

Our study grouped 172 countries based on their World Bank good governance indicators into four clusters using K-means clustering. Our analysis suggests that countries with the highest rates of COVID-19 vaccination are those ranking high in effectiveness in regulatory quality, voice and accountability, and government effectiveness. These countries include, for example, Israel, the United Arab Emirates, the United States, and the United Kingdom. Countries in clusters with poor governance indicators also did not start any COVID-19 vaccinations at the time of our study.

Our findings are consistent with prior research that investigated the government's effectiveness in controlling the COVID-19 pandemic. A recent study found that Covid‑19 mortality.

is negatively associated with government effectiveness and noted that government effectiveness remains critical for the COVID-19 pandemic^8^. Also, effective governments may respond to the COVID-19 pandemic with efficient and coordinated logistical planning including increased COVID-19 testing and hospital capacity, and monitoring and supplying of personal protective equipment^8,17^. Additionally, another study emphasized that effectively managing and treating COVID-19 patients was correlated with the quality of government^18^. Other studies focused on governments' early or strict implementation of nonpharmaceutical interventions in controlling the COVID-19 pandemic. They found that government policies such as business closures and stay-at-home orders successfully mitigated the spread of the COVID-19 virus^9,10,19^. However, the effectiveness of these nonpharmaceutical interventions is also likely contingent on the responsive or willingness of a nation's population to adhere to them. In our study, regulatory quality had the strongest predictive power in explaining variation in vaccination rates across countries. As defined by the World Bank, regulatory quality "captures perceptions of the ability of the government to formulate and implement sound policies and regulations".

Across the 172 countries in our dataset, Israel, the United Arab Emirates, and Bahrain have the highest per-capita rates of COVID-19 vaccination (Table A3). All three are relatively newly established nations, being founded in the twentieth century, and they rank relatively highly in World Bank indicators of government effectiveness and regulatory quality as well as rule of law. However, the United Arab Emirates and Bahrain rank low on the voice and accountability indicator. Also interesting is that both Israel and Bahrain rank low on political stability. In our machine learning analysis, there was a significant decrease in relative gain from the government effectiveness indicator to the 4th ranked indicator, political stability (Fig. 4).

By contrast, despite the fact that New Zealand, Australia, Japan, and South Korea also have very high governance indicators, these countries did not prioritize nationwide vaccinations and are lagging behind other OECD countries. This is surprising because South Korea, New Zealand, and Australia had very effective mitigation policies against COVID-19 (e.g., self-isolation on arrival, border closure, and strict lockdown policies), and they had low rates of or near elimination of community transmission of the virus^20,21^. New Zealand, for example, virtually eliminated community transmission using very strict lockdowns, but, as of the time of our study, had not started vaccinating their population. Australia is similar to New Zealand in good government indicators, but also did not begin vaccinations during our study period. In Australia's case, this may be partly due to the abandonment of an Australian-developed vaccine, which caused false positive HIV tests. Japan is an example of where, although it ranks relatively high on regulatory quality as well as rule of law and government effectiveness, it did not adopt strict pandemic mitigation measures compared to its regional neighbors, and its conservative vaccine approval process delayed approval of any COVID-19 vaccine until more than six weeks after the World Health Organization had approved the Pfizer-BioNTech vaccine for emergency use^22^. South Korea also began COVID-19 vaccinations in late February as one of the last OECD countries to start nationwide vaccinations, and this may have been associated with widespread public skepticism of vaccine safety^23^. Also interesting in our data is that, despite having very strict pandemic mitigation efforts, China is lagging other countries in vaccinating its population, although it has developed multiple vaccines. However, China has poor World Bank governance indicators, which is correlated with slow vaccination response in our analysis.

We also found a few countries that were exceptional in our analysis. Spain and Belgium rank relatively high across governance indicators. Spain vaccinated over 2.19% of its population at the time of our study. Belgium had comparable rates of COVID-19 cases and higher rates of COVID-19 deaths, but has nearly half the vaccination rate as Spain (Table A3). Complicating vaccination efforts in Europe has been a centralized and delayed response to securing vaccination supplies at the European Union level^24^. Thus, although several EU nations like Belgium, the Netherlands, France, Germany, Sweden and Switzerland have superior governance indicators, they have relatively low rates of vaccination of their populations. However, smaller EU nations such as Malta, Denmark, Slovenia, and Ireland rank in the top ten countries in terms of vaccination rates.

This study should be interpreted in the context of certain limitations. We conducted our analyses based on World Bank good governance indicators. The validity of governance measures is uncertain and they may be over-generalized given substantial variation in governmental structure and policies across countries; however, the World Bank indicators are widely used and authoritative^25^. Also, our study is based on the initiation of the COVID-19 vaccination, and our findings will need to be re-evaluated over time as vaccinations continue to be disseminated globally. There may be other societal factors correlated with governance indicators that are important in determining acceptance, early adoption, and effective distribution of COVID-19 vaccinations. More research is needed to identify and characterize these additional societal factors. Finally, there may be substantial variation in governance indicators across regions within countries that may enhance or inhibit governance and pandemic response at the national level.

## Conclusions

We explore the relationship of World Bank governance indicators in predicting COVID-19 vaccination status across 172 countries. Our analysis suggests that good governance indicators, in particular, regulatory quality, voice and accountability, and government effectiveness, are the most important indicators in predicting COVID-19 vaccinations across countries. Using a machine learning approach, our study identified national-level factors that were predictive of adoption and roll-out of the COVID-19 vaccine. Although improved governance is unlikely to be practical or feasible in a reasonable time period to address the ongoing pandemic, our findings call for a coordinated global effort led by the World Health Organization and wealthier nations to aid with the supply and distribution of vaccines to those countries that have less effective governance structures. Not doing so risks creating persistent "hot spots" of endemic COVID-19 spread, which will substantially increase the opportunities of new, increasingly challenging variants to arise, ultimately impacting not only the populations of these countries but also those throughout the world.

## Supplementary Information

Supplementary Information.

## Data Availability

Data are publicly available.
