# Supplementary material for: The role of good governance in the race for global vaccination during the COVID-19 pandemic
Source: Sci Rep. 2021 Nov 17;11:22440. doi: 10.1038/s41598-021-01831-0 (PMC8599507; doi:10.1038/s41598-021-01831-0)
Supplement: Supplementary file 1 — Supplementary Information. [file 41598_2021_1831_MOESM1_ESM.pdf]

Supplementary

The Role of Good Governance in the Race for Global Vaccination during the COVID-19 Pandemic

Appendix

Figure A1. Clustering Results of 172 Countries' Good Governance Indicators Using the K-means Clustering Algorithm and Visualized Using Principal Components Analysis

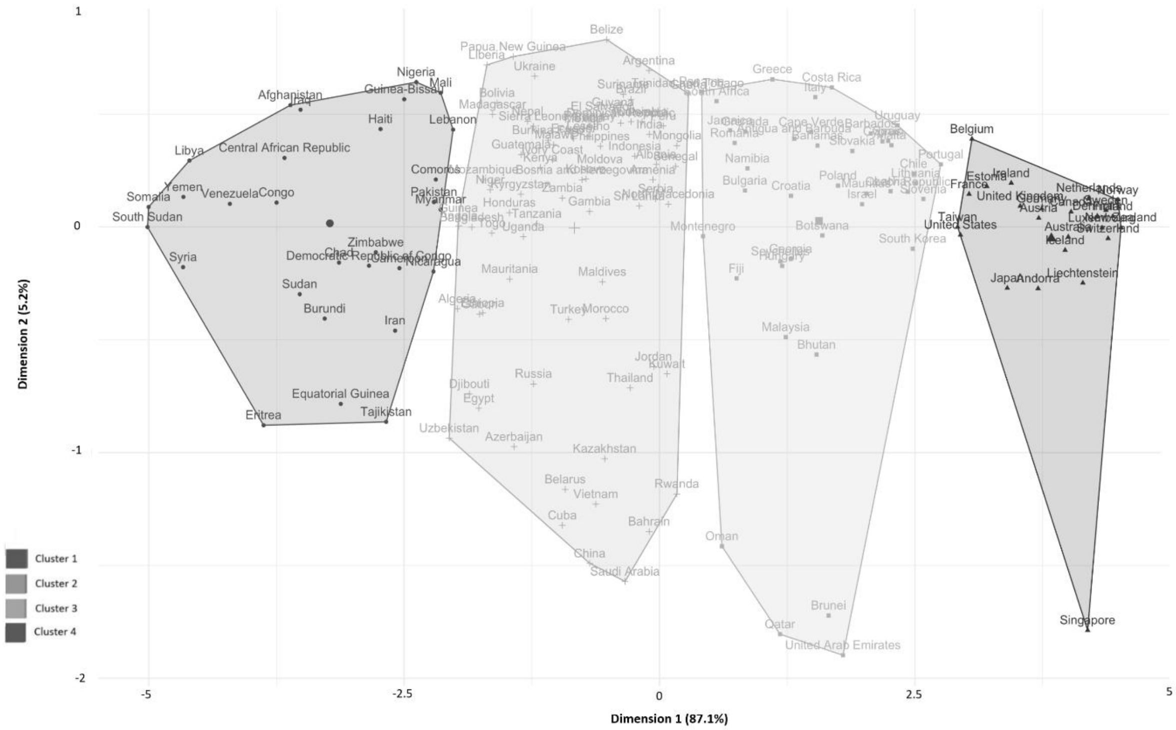

Cluster 1: Afghanistan, Antigua and Barbuda, Burundi, Cameroon, Central African Republic, Chad, Comoros, Congo, Democratic Republic of Congo, Equatorial Guinea, Eritrea, Guinea-Bissau, Haiti, Iran, Iraq, Lebanon, Libya, Mali, Myanmar, Nicaragua, Nigeria, Pakistan, Somalia, South Sudan, Sudan, Syria, Tajikistan, Venezuela, Yemen, Zimbabwe, **Cluster 2:** Albania, Algeria, Angola, Argentina, Armenia, Azerbaijan, Bahrain, Bangladesh, Belarus, Belize, Benin, Bolivia, Bosnia and Herzegovina, Brazil, Burkina Faso, China, Colombia, Cuba, Djibouti, Dominican Republic, Ecuador, Egypt, El Salvador, Ethiopia, Gabon, Gambia, Ghana, Guatemala, Guinea, Guyana, Honduras, India, Indonesia, Ivory Coast, Jordan, Kazakhstan, Kenya, Kosovo, Kuwait, Kyrgyzstan, Lesotho, Liberia, Madagascar, Malawi, Maldives, Mauritania, Mexico, Moldova, Mongolia, Morocco, Mozambique, Nepal, Niger, North, Macedonia, Papua New Guinea, Paraguay, Peru, Philippines, Russia, Rwanda, Saudi Arabia, Senegal, Serbia, Sierra Leone, Sri Lanka, Suriname, Tanzania, Thailand, Togo, Trinidad and Tobago, Tunisia, Turkey, Uganda, Ukraine, Uzbekistan, Vietnam, ,Zambia, **Cluster 3:** Bahamas, Barbados, Bhutan, Botswana, Brunei, Bulgaria, Cape Verde, Chile, Costa Rica, Croatia, Cyprus, Czech Republic, Fiji, Georgia, Greece, Grenada, Hungary, Israel, Italy, Jamaica, Latvia, Lithuania, Malaysia, Malta, Mauritius, Montenegro, Namibia, Oman, Panama, Poland, Portugal, Qatar, Romania, Seychelles, Slovakia, Slovenia, South Africa, South Korea, Spain, United Arab Emirates, Uruguay, **Cluster**

4: Andorra, Australia, Austria, Belgium, Canada, Denmark, Estonia, Finland, France, Germany, Iceland, Ireland, Japan, Liechtenstein, Luxembourg, Netherlands, New Zealand, Norway, Singapore, Sweden, Switzerland, Taiwan, United Kingdom, United States. Countries with no available data are shown in gray. The maps were generated using RStudio 4.0.2 (R Core Team, 2020). Downloaded from: https://www.rstudio.com/products/rstudio/download/

Table A1. The World Bank's Worldwide Governance Indicators Definition of Good Governance

Indicator	Definition
**Control of Corruption**	Control of Corruption captures perceptions of the extent to which public power is exercised for private Gain, including both petty and grand forms of corruption, as well as "capture" of the state by elites and private interests. Estimate gives the country's score on the aggregate indicator, in units of a standard normal distribution, i.e. ranging from approximately -2.5 to 2.5.
**Government Effectiveness**	Government Effectiveness captures perceptions of the Quality of public services, the quality of the civil service and the degree of its independence from political pressures, the Quality of policy formulation and implementation, and the credibility of the government's commitment to such policies. Estimate gives the country's score on the aggregate indicator, in units of a standard normal distribution, i.e. ranging from approximately -2.5 to 2.5.
**Political Stability**	Political Stability and Absence of Violence/Terrorism measures perceptions of the likelihood of political instability and/or politically-motivated violence, including terrorism. Estimate gives the country's score on the aggregate indicator, in units of a standard normal distribution, i.e. ranging from approximately -2.5 to 2.5.
**Regulatory Quality**	Regulatory Quality captures perceptions of the ability of the government to formulate and implement sound policies and regulations that permit and promote private sector development. Estimate gives the country's score on the aggregate indicator, in units of a standard normal distribution, i.e. ranging from approximately -2.5 to 2.5.
**Rule of Law**	Rule of Law captures perceptions of the extent to which agents have confidence in and abide by the rules of society, and in particular the Quality of contract enforcement, property rights, the police, and the courts, as well as the likelihood of crime and violence. Estimate gives the country's score on the aggregate indicator, in units of a standard normal distribution, i.e. ranging from approximately -2.5 to 2.5.
**Voice and Accountability**	Voice and Accountability captures perceptions of the extent to which a country's citizens are able to participate in selecting their government, as well as freedom of expression, freedom of association, and a free media. Estimate gives the country's score on the aggregate indicator, in units of a standard normal distribution, i.e. ranging from approximately -2.5 to 2.5.

Table A2. Descriptive Statistics of Good Governance Indicators and COVID-19 in 172 Countries

Feature	Minimum	Median	Mean	Maximum	Standard Deviation
Control of Corruption	-1.8	-0.3	-0.1	2.2	1.0
Government Effectiveness	-2.5	-0.1	0.0	2.2	1.0
Regulatory Quality	-2.4	-0.2	0.0	2.2	1.0
Rule of Law	-2.4	-0.3	-0.1	2.0	1.0
Voice and Accountability	-2.2	0.0	-0.1	1.7	1.0
Political Stability	-2.8	-0.1	-0.2	1.7	1.0
COVID-19 Cases per Million by January	8.5	9,541	19,486	120,469	23,053
COVID-19 Cases per Million by March	8.5	11,233	26,433	147,945	32,247
COVID-19 Deaths per Million by January	0.2	99	355	1,775	442
COVID-19 Deaths per Million by March	0.2	152	472	2,467	606
Vaccinations per Hundred People					
One Month After COVID-19 Vaccination	0	0	0.9	36.8	3.5
Three Months After COVID-19 Vaccination	0	1.6	9.1	115.9	16.8

Table A3. Ranking of COVID-19 Vaccinations per Hundred People, Cases per Million, and Deaths per Million with World Bank Governance Indicators by Country and Territory

Country	Cluster	Vaccinations per Hundred People One Month After COVID-19 Vaccination	Vaccination Status One Month After COVID-19 Vaccination	Vaccinations per Hundred People Three Months After COVID-19 Vaccination	Vaccination Status Three Months After COVID-19 Vaccination	COVID-19 Cases per Million by January	COVID-19 Deaths per Million by January	Control of Corruption	Government Effectiveness	Regulatory Quality	Rule of Law	Voice and Accountability	Political Stability
Israel	3	36.8	1	116	1	66,529	483	0.81	1.33	1.28	1.05	0.69	-0.78
United Arab Emirates	3	21.9	1	84	1	26,665	77	1.11	1.38	0.98	0.84	-1.12	0.70
Seychelles	3	13.4	1	102	1	7,749	20	0.97	0.52	-0.15	0.17	0.34	0.69
Bahrain	2	8.47	1	45	1	57,930	215	-0.01	0.30	0.49	0.49	-1.41	-0.66
United Kingdom	4	8.01	1	52.5	1	51,790	1,377	1.77	1.44	1.63	1.60	1.26	0.52
United States	4	4.94	1	44.9	1	73,909	1,249	1.22	1.49	1.35	1.46	0.97	0.30
Malta	3	3.47	1	44.7	1	36,529	553	0.24	0.86	0.96	0.95	1.11	1.09
Denmark	4	3.2	1	19.6	1	33,180	323	2.11	1.94	1.57	1.90	1.58	1.01
Slovenia	3	2.65	1	17.7	1	73,524	1,567	0.91	1.08	1.01	1.12	1.01	0.82
Ireland	4	2.47	1	17	1	36,317	561	1.46	1.28	1.60	1.39	1.34	0.97
Lithuania	3	2.44	1	18.9	1	63,407	927	0.68	1.04	1.16	1.02	1.02	0.84
Spain	3	2.19	1	17.2	1	51,595	1,169	0.65	1.00	1.05	0.98	1.09	0.32
Italy	3	2.17	1	17	1	39,929	1,384	0.24	0.46	0.95	0.28	0.97	0.46
Iceland	4	1.82	1	21.4	1	17,509	85	1.71	1.52	1.37	1.77	1.33	1.66
Germany	4	1.78	1	16.4	1	25,072	597	1.90	1.59	1.72	1.62	1.34	0.58
Portugal	3	1.74	1	16.6	1	57,039	928	0.76	1.15	0.97	1.14	1.24	1.13
Slovakia	3	1.61	1	18	1	41,903	684	0.33	0.67	1.01	0.56	0.91	0.78
Romania	3	1.6	1	16.1	1	36,434	909	-0.13	-0.28	0.46	0.36	0.49	0.53
Czech Republic	3	1.56	1	16	1	84,894	1,384	0.51	0.89	1.25	1.05	0.94	0.95
Poland	3	1.56	1	16.6	1	38,332	902	0.60	0.60	1.01	0.45	0.70	0.52
Estonia	4	1.54	1	19.7	1	29,056	263	1.54	1.17	1.59	1.28	1.21	0.64
Belgium	4	1.5	1	16.4	1	59,040	1,775	1.55	1.03	1.29	1.36	1.37	0.48
Hungary	3	1.43	1	29.5	1	36,671	1,202	0.00	0.50	0.60	0.49	0.22	0.73
Austria	4	1.41	1	18.3	1	44,201	804	1.55	1.49	1.46	1.88	1.33	0.98
Croatia	3	1.32	1	11.6	1	55,185	1,148	0.13	0.41	0.59	0.37	0.53	0.76
Canada	4	1.29	1	15.1	1	19,351	492	1.77	1.73	1.72	1.76	1.46	1.03
Norway	4	1.28	1	17.9	1	11,047	100	2.07	1.86	1.80	1.98	1.69	1.19
Turkey	2	1.25	1	18.8	1	28,530	290	-0.29	0.05	-0.01	-0.28	-0.81	-1.34
Sweden	4	1.19	1	16	1	53,268	1,069	2.12	1.83	1.80	1.91	1.59	1.05

Cyprus	3	1.17	1	14.8	1	33,648	201	0.60	0.99	1.01	0.76	1.08	0.52
Finland	4	1.12	1	17.7	1	7,430	114	2.15	1.93	1.85	2.02	1.59	0.91
France	4	1.05	1	16.7	1	46,323	1,100	1.30	1.38	1.44	1.41	1.14	0.31
Greece	3	1.05	1	16.3	1	14,389	532	-0.01	0.41	0.53	0.20	0.94	0.29
China	2	1.04	1	8.32	1	68	3	-0.32	0.52	-0.24	-0.27	-1.61	-0.24
Luxembourg	4	0.99	1	15.3	1	78,604	898	2.11	1.73	1.70	1.79	1.52	1.36
Latvia	3	0.98	1	7.39	1	30,648	547	0.48	1.11	1.19	1.01	0.88	0.45
Saudi Arabia	2	0.85	1	12.7	1	10,501	182	0.27	0.31	-0.07	0.17	-1.62	-0.43
Russia	2	0.69	1	7.79	1	24,635	454	-0.83	0.15	-0.43	-0.72	-1.10	-0.54
Serbia	2	0.66	1	35.9	1	55,469	560	-0.45	0.02	0.11	-0.12	0.03	-0.09
Netherlands	4	0.58	1	13.9	1	54,865	779	2.00	1.80	1.86	1.81	1.56	0.86
Costa Rica	3	0.58	1	7.55	1	36,849	489	0.72	0.42	0.50	0.54	1.13	0.46
Oman	3	0.58	1	2.97	1	25,911	297	0.45	0.26	0.29	0.55	-1.14	0.59
Argentina	2	0.55	1	8.61	1	40,528	1,023	-0.07	-0.09	-0.49	-0.43	0.60	-0.12
Mexico	2	0.39	1	6.09	1	13,099	1,120	-0.82	-0.16	0.10	-0.66	0.02	-0.71
Bulgaria	3	0.35	1	6.8	1	30,713	1,245	-0.16	0.34	0.53	0.04	0.38	0.54
Chile	3	0.32	1	54.8	1	35,611	920	1.09	1.06	1.22	1.07	1.02	0.21
Singapore	4	0.11	1	22.5	1	10,119	5	2.16	2.22	2.16	1.88	-0.18	1.53
India	2	0.06	1	4.72	1	7,689	111	-0.23	0.17	-0.16	-0.03	0.29	-0.70
Albania	2	0.02	1	4.15	1	24,059	449	-0.53	-0.06	0.27	-0.41	0.15	0.12
Panama	3	0.01	1	8.59	1	70,404	1,138	-0.58	0.07	0.36	-0.12	0.62	0.31
Brazil	2	0.01	1	9.01	1	40,639	1,001	-0.33	-0.19	-0.18	-0.18	0.34	-0.55
Andorra	4	0	0	12	1	120,469	1,191	1.23	1.91	1.23	1.58	1.14	1.62
Montenegro	3	0	0	3.57	1	90,085	1,207	-0.03	0.16	0.37	0.10	0.03	0.01
Liechtenstein	4	0	0	14.4	1	63,062	1,311	1.97	1.70	1.54	1.68	1.26	1.63
Georgia	3	0	0	0.2	1	62,653	749	0.67	0.83	1.12	0.31	0.20	-0.45
Switzerland	4	0	0	17.8	1	58,341	1,031	1.98	1.95	1.66	1.91	1.53	1.34
Armenia	2	0	0	0	0	55,757	1,018	-0.18	-0.07	0.25	-0.13	0.05	-0.51
Qatar	3	0	0	28.3	1	51,370	86	0.85	0.71	0.68	0.73	-1.29	0.70
North Macedonia	2	0	0	0.37	1	42,941	1,315	-0.41	0.00	0.51	-0.24	0.00	-0.05
Lebanon	1	0	0	3.17	1	38,774	305	-1.16	-0.83	-0.43	-0.86	-0.49	-1.64
Colombia	2	0	0	4.41	1	38,460	979	-0.23	0.07	0.40	-0.42	0.23	-0.90
Moldova	2	0	0	1	1	38,205	818	-0.62	-0.38	0.01	-0.37	-0.09	-0.38
Kuwait	2	0	0	14.2	1	37,293	223	-0.13	0.02	0.06	0.22	-0.63	0.20
Bosnia and Herzegovina	2	0	0	0	0	36,185	1,378	-0.61	-0.63	-0.19	-0.23	-0.20	-0.40
Peru	2	0	0	2.59	1	32,549	1,184	-0.45	-0.07	0.56	-0.49	0.29	-0.14
Jordan	2	0	0	4.01	1	31,109	410	0.13	0.10	0.03	0.14	-0.67	-0.36
Belize	2	0	0	5.55	1	29,279	719	-0.20	-0.68	-0.54	-0.78	0.53	0.07
Kosovo	2	0	0	0	0	29,187	735	-0.56	-0.35	-0.35	-0.39	-0.13	-0.39
Ukraine	2	0	0	0.57	1	27,687	509	-0.71	-0.30	-0.26	-0.70	0.06	-1.52
Maldives	2	0	0	44	1	27,217	91	-0.26	-0.19	-0.48	-0.41	-0.44	0.01
Belarus	2	0	0	0.71	1	24,393	170	-0.06	-0.18	-0.54	-0.79	-1.40	0.29
Cape Verde	3	0	0	0.39	1	23,785	218	0.87	0.29	-0.22	0.52	0.97	0.88
South Africa	3	0	0	0.44	1	23,090	655	0.08	0.37	0.16	-0.08	0.67	-0.22

Azerbaijan	2	0	0	5.03	1	22,490	300	-0.87	-0.14	-0.23	-0.58	-1.49	-0.68
Bahamas	3	0	0	1.78	1	20,534	445	1.18	0.49	-0.05	0.08	0.93	0.83
Dominican Republic	2	0	0	8.4	1	18,264	228	-0.76	-0.36	-0.05	-0.35	0.20	0.02
Paraguay	2	0	0	0.64	1	17,448	358	-0.83	-0.53	-0.20	-0.56	0.08	0.00
Bolivia	2	0	0	2.61	1	16,598	836	-0.74	-0.70	-0.99	-1.12	-0.09	-0.62
Libya	1	0	0	0	0	16,172	250	-1.60	-1.92	-2.35	-1.85	-1.46	-2.57
Iran	1	0	0	0.15	1	16,053	679	-1.05	-0.55	-1.42	-0.75	-1.37	-1.70
Tunisia	2	0	0	0.45	1	15,939	501	-0.08	-0.10	-0.44	0.06	0.28	-0.83
Iraq	1	0	0	0.07	1	15,181	322	-1.34	-1.34	-1.18	-1.72	-0.95	-2.56
Honduras	2	0	0	0.43	1	13,822	344	-0.81	-0.61	-0.49	-1.01	-0.55	-0.53
Ecuador	2	0	0	1.39	1	13,281	818	-0.50	-0.40	-0.82	-0.58	0.06	-0.19
Suriname	2	0	0	4.44	1	13,267	249	-0.39	-0.59	-0.68	-0.06	0.39	0.11
Kyrgyzstan	2	0	0	0	0	12,788	213	-0.95	-0.68	-0.35	-0.89	-0.46	-0.41
Morocco	2	0	0	21.7	1	12,531	218	-0.28	-0.12	-0.21	-0.14	-0.63	-0.37
Namibia	3	0	0	0.06	1	12,300	118	0.37	0.10	-0.11	0.31	0.54	0.53
Kazakhstan	2	0	0	0.59	1	11,691	157	-0.32	0.12	0.14	-0.43	-1.21	-0.08
Uruguay	3	0	0	19.4	1	9,872	97	1.25	0.70	0.51	0.62	1.26	1.05
Nepal	2	0	0	5.49	1	9,209	68	-0.67	-1.05	-0.70	-0.54	-0.13	-0.47
Guyana	2	0	0	4.16	1	8,919	216	-0.09	-0.39	-0.62	-0.43	0.28	-0.24
Guatemala	2	0	0	0.6	1	8,447	298	-0.90	-0.68	-0.22	-1.05	-0.31	-0.55
El Salvador	2	0	0	1.54	1	7,930	234	-0.55	-0.47	0.02	-0.76	0.14	-0.13
Botswana	3	0	0	0	0	7,922	37	0.71	0.43	0.37	0.50	0.53	1.01
Djibouti	2	0	0	0	0	5,985	62	-0.86	-0.71	-0.77	-0.91	-1.41	-0.34
Trinidad and Tobago	2	0	0	0.07	1	5,309	95	-0.19	0.10	-0.15	-0.12	0.62	0.06
Malaysia	3	0	0	2.16	1	5,233	19	0.25	1.00	0.67	0.59	-0.04	0.11
Jamaica	3	0	0	1.11	1	4,892	112	-0.06	0.50	0.17	-0.31	0.64	0.41
Philippines	2	0	0	0.46	1	4,617	92	-0.57	0.05	0.01	-0.48	0.03	-0.88
Gabon	2	0	0	0.01	1	4,547	30	-0.94	-0.90	-0.96	-0.73	-1.03	-0.18
Venezuela	1	0	0	0.05	1	4,279	39	-1.51	-1.66	-2.36	-2.32	-1.41	-1.45
Barbados	3	0	0	22.2	1	4,023	31	1.26	0.63	0.41	0.36	1.13	0.94
Equatorial Guinea	1	0	0	0.87	1	3,824	61	-1.72	-1.34	-1.52	-1.42	-1.86	-0.19
Mauritania	2	0	0	0	0	3,487	88	-0.86	-0.50	-0.76	-0.58	-0.78	-0.54
Indonesia	2	0	0	4.27	1	3,436	98	-0.42	0.18	-0.09	-0.34	0.16	-0.48
Lesotho	2	0	0	0	0	3,284	48	-0.03	-0.83	-0.54	-0.38	0.03	-0.35
Bangladesh	2	0	0	3.26	1	3,216	48	-0.99	-0.74	-0.93	-0.64	-0.72	-0.92
Japan	4	0	0	0.79	1	2,740	38	1.48	1.59	1.33	1.54	0.96	1.04
Sri Lanka	2	0	0	4.26	1	2,577	13	-0.32	-0.11	-0.18	-0.01	-0.04	-0.23
Myanmar	1	0	0	0.7	1	2,494	55	-0.63	-1.15	-0.76	-1.06	-0.84	-1.26
Pakistan	1	0	0	0.36	1	2,386	51	-0.85	-0.68	-0.64	-0.67	-0.84	-2.25
Algeria	2	0	0	0.17	1	2,385	65	-0.62	-0.52	-1.30	-0.82	-1.04	-1.00
Uzbekistan	2	0	0	0	0	2,335	19	-1.05	-0.51	-0.99	-1.05	-1.61	-0.27
Zambia	2	0	0	0	0	2,227	32	-0.64	-0.68	-0.55	-0.46	-0.29	-0.10
Comoros	1	0	0	0	0	2,223	62	-1.02	-1.67	-1.12	-1.09	-0.69	-0.15
Zimbabwe	1	0	0	0.62	1	1,979	59	-1.24	-1.21	-1.46	-1.26	-1.14	-0.92

Antigua and Barbuda	1	0	0	27	1	1,940	61	0.28	0.00	0.49	0.41	0.76	0.96
Ghana	2	0	0	1.61	1	1,880	12	-0.08	-0.21	-0.11	0.05	0.58	0.10
Kenya	2	0	0	0.3	1	1,849	32	-0.78	-0.38	-0.28	-0.45	-0.29	-1.12
Cuba	2	0	0	0	0	1,688	16	0.04	-0.17	-1.49	-0.32	-1.42	0.61
Gambia	2	0	0	0.22	1	1,630	53	-0.29	-0.63	-0.66	-0.37	-0.22	0.11
Egypt	2	0	0	0	0	1,553	85	-0.67	-0.42	-0.83	-0.42	-1.43	-1.07
South Korea	3	0	0	1.74	1	1,442	26	0.76	1.38	1.07	1.19	0.77	0.48
Senegal	2	0	0	1.56	1	1,412	33	0.05	-0.06	-0.11	-0.19	0.26	0.06
Congo	1	0	0	0	0	1,397	21	-1.54	-1.63	-1.51	-1.79	-1.37	-1.81
Tajikistan	1	0	0	0	0	1,395	9	-1.32	-1.05	-1.01	-1.23	-1.83	-0.58
Afghanistan	1	0	0	0.14	1	1,394	60	-1.40	-1.46	-1.12	-1.71	-0.99	-2.65
Guinea-Bissau	1	0	0	0	0	1,275	23	-1.45	-1.51	-1.23	-1.26	-0.46	-0.56
Grenada	3	0	0	8.73	1	1,235	9	0.34	-0.14	-0.31	0.18	0.71	0.96
Ethiopia	2	0	0	0	0	1,148	18	-0.41	-0.63	-0.89	-0.47	-1.05	-1.28
Australia	4	0	0	2.63	1	1,127	36	1.81	1.57	1.87	1.73	1.32	1.09
Bhutan	3	0	0	51.2	1	1,102	1	1.62	0.31	-0.33	0.59	0.10	1.09
Guinea	2	0	0	0.43	1	1,082	6	-0.90	-0.78	-0.77	-1.21	-0.75	-0.83
Cameroon	1	0	0	0	0	1,055	17	-1.21	-0.81	-0.83	-1.12	-1.19	-1.56
Central African Republic	1	0	0	0	0	1,030	13	-1.23	-1.75	-1.37	-1.73	-1.20	-2.18
Ivory Coast	2	0	0	0.15	1	970	5	-0.53	-0.48	-0.24	-0.57	-0.22	-0.96
Haiti	1	0	0	0	0	957	21	-1.34	-2.02	-1.26	-0.97	-0.73	-0.78
Mozambique	2	0	0	0.15	1	941	9	-0.80	-0.82	-0.72	-1.02	-0.51	-0.75
Nicaragua	1	0	0	0	0	937	25	-1.12	-0.77	-0.69	-1.18	-1.08	-1.03
Rwanda	2	0	0	2.69	1	916	12	0.56	0.19	0.08	0.08	-1.08	0.12
Uganda	2	0	0	0.2	1	844	7	-1.17	-0.59	-0.37	-0.31	-0.62	-0.65
Malawi	2	0	0	0.7	1	776	18	-0.78	-0.75	-0.70	-0.33	-0.09	-0.27
Syria	1	0	0	0	0	761	49	-1.69	-1.71	-1.76	-2.08	-1.98	-2.73
Madagascar	2	0	0	0	0	661	10	-1.01	-1.14	-0.73	-1.01	-0.22	-0.25
Sudan	1	0	0	0	0	650	37	-1.37	-1.62	-1.67	-1.14	-1.64	-1.67
Angola	2	0	0	0.4	1	581	14	-1.05	-1.12	-0.89	-1.05	-0.78	-0.31
Nigeria	1	0	0	0.35	1	556	7	-1.09	-1.09	-0.86	-0.90	-0.41	-1.93
Togo	2	0	0	0.51	1	539	9	-0.75	-0.92	-0.68	-0.59	-0.72	-0.81
Eritrea	1	0	0	0	0	539	2	-1.39	-1.76	-2.27	-1.60	-2.19	-0.72
Mongolia	2	0	0	9.16	1	483	1	-0.44	-0.19	-0.01	-0.27	0.35	0.64
New Zealand	4	0	0	1.42	1	470	5	2.17	1.67	1.88	1.88	1.57	1.51
Burkina Faso	2	0	0	0	0	457	5	-0.19	-0.76	-0.38	-0.43	-0.18	-1.19
Mauritius	3	0	0	0.3	1	437	8	0.32	0.87	1.00	0.76	0.81	0.82
Brunei	3	0	0	0	0	398	7	0.80	1.32	0.63	0.61	-0.95	1.17
Mali	1	0	0	0	0	390	16	-0.70	-1.06	-0.57	-0.83	-0.41	-2.15
Sierra Leone	2	0	0	0.32	1	380	10	-0.41	-1.13	-0.88	-0.77	-0.10	-0.10
Liberia	2	0	0	0	0	376	17	-0.88	-1.38	-0.98	-1.00	-0.03	-0.23
South Sudan	1	0	0	0	0	337	6	-1.77	-2.45	-2.05	-1.97	-1.97	-2.56
Somalia	1	0	0	0	0	298	8	-1.71	-2.24	-2.20	-2.35	-1.84	-2.38
Benin	2	0	0	0	0	293	4	-0.32	-0.44	-0.38	-0.66	0.09	-0.35

**Democratic Republic of Congo**	1	0	0	0	0	238	7	-1.41	-1.39	-1.30	-1.15	-1.26	-0.89
**Chad**	1	0	0	0	0	183	7	-1.42	-1.57	-1.12	-1.28	-1.42	-1.34
**Thailand**	2	0	0	0.26	1	183	1	-0.41	0.36	0.12	0.10	-0.83	-0.54
**Niger**	2	0	0	0	0	176	6	-0.55	-0.80	-0.67	-0.53	-0.57	-1.40
**Burundi**	1	0	0	0	0	111	0	-1.46	-1.33	-0.95	-1.43	-1.71	-1.65
**Papua New Guinea**	2	0	0	0	0	93	1	-0.99	-0.81	-0.54	-0.80	0.07	-0.83
**Yemen**	1	0	0	0	0	71	21	-1.68	-2.28	-1.66	-1.77	-1.77	-2.77
**Fiji**	3	0	0	0	0	61	2	0.56	0.20	-0.22	-0.03	0.09	0.87
**Taiwan**	4	0	0	0.06	1	37	0	1.05	1.44	1.40	1.14	1.00	0.72
**Vietnam**	2	0	0	0.05	1	16	0	-0.51	0.04	-0.26	-0.02	-1.38	0.13
**Tanzania**	2	0	0	0	0	9	0	-0.39	-0.88	-0.64	-0.58	-0.50	-0.36

Darker colors shows higher rates and numbers. Country's good governance score ranging from approximately -2.5 to 2.5 (higher score shows the indicator has a better situation and is shown with darker green and lower score shows the indicator has a worse situation and is shown with darker brown). Countries that started COVID-19 vaccination within the first month of vaccine availability are labeled "1" in the Vaccination Status column.

Table A4. XGBoost Classification Confusion Matrix

One Month After COVID-19 Vaccination

		Actual	
		**0**	**1**
**Prediction**	**0**	105	17
	**1**	16	34

Three Months After COVID-19 Vaccination

		Actual	
		**0**	**1**
**Prediction**	**0**	20	12
	**1**	21	119

Positive class is labeled as "1"

Table A5. XGBoost Classification Model and Prediction Evaluation

Measure	One Month After COVID-19 Vaccination	Three Months After COVID-19 Vaccination
Accuracy	0.81	0.81
95% CI	(0.74, 0.86)	(0.74, 0.86)
Sensitivity	0.67	0.91
Specificity	0.87	0.49
Pos Predictive Value	0.68	0.85
Neg Predictive Value	0.86	0.63
Precision	0.68	0.85
F1	0.67	0.88
Prevalence	0.30	0.76
Balanced Accuracy	0.77	0.70

Table A6. XGBoost Feature (Indicator) Relative Importance Matrix for Good Governance

Feature	One Month After COVID-19 Vaccination			Three Months After COVID-19 Vaccination		
	Gain	Cover	Frequency	Gain	Cover	Frequency
Regulatory Quality	22.7	16.8	16.7	16.3	16.4	16.1
Voice and Accountability	22.2	24	24	21.9	22	22
Government Effectiveness	20.9	14.3	13.8	16.1	15.2	14.8
Political Stability	14.8	18.7	18.8	19	17.9	18.2
Rule of Law	11.4	14	14.2	13.8	14	14.5
Control of Corruption	8	12.2	12.6	13	13.7	14.2
Sum %	100	100	100	100	100	100
